# Mesenchymal-Stem-Cell-Based Therapy against Gliomas

**DOI:** 10.3390/cells13070617

**Published:** 2024-04-02

**Authors:** Sisa M. Santillán-Guaján, Mehdi H. Shahi, Javier S. Castresana

**Affiliations:** 1 Department of Biochemistry and Genetics, University of Navarra School of Sciences, 31008 Pamplona, Spain; ssantillanguajan@gmail.com; 2 Interdisciplinary Brain Research Centre, Faculty of Medicine, Aligarh Muslim University, Aligarh 202002, India; mehdihayat.md@amu.ac.in

**Keywords:** mesenchymal stem cells, glioblastoma, CAR-T, nanoparticles, tropism, exosomes

## Abstract

Glioblastoma is the most aggressive, malignant, and lethal brain tumor of the central nervous system. Its poor prognosis lies in its inefficient response to currently available treatments that consist of surgical resection, radiotherapy, and chemotherapy. Recently, the use of mesenchymal stem cells (MSCs) as a possible kind of cell therapy against glioblastoma is gaining great interest due to their immunomodulatory properties, tumor tropism, and differentiation into other cell types. However, MSCs seem to present both antitumor and pro-tumor properties depending on the tissue from which they come. In this work, the possibility of using MSCs to deliver therapeutic genes, oncolytic viruses, and miRNA is presented, as well as strategies that can improve their therapeutic efficacy against glioblastoma, such as CAR-T cells, nanoparticles, and exosomes.

## 1. Introduction

Glioblastoma is the most common and lethal primary brain tumor of the CNS. Due to its invasive and infiltrative growth pattern, its complete eradication through surgery is practically impossible. Furthermore, its high resistance to radiotherapy and concomitant chemotherapy with temozolomide mean its median survival rate remains at 15 months [1,2]. Intratumor heterogeneity conditions different degrees of sensitivity to treatment. Furthermore, the blood–brain barrier that limits drug access to the tumor site, the tumor microenvironment, and the rapid development of resistant phenotypes are responsible for the failure of several targeted therapies. One treatment option to overcome these limitations might be the use of mesenchymal stem cells (MSCs).

MSCs are multipotent cells capable of self-renewal and differentiation into multiple cell types. They are currently used in tissue regeneration and immune disorders, but a better understanding of the biology of MSCs has made it possible to explore their potential as a new therapeutic tool against brain tumors [3,4]. De Melo et al. [3] treated a U-87 brain tumor cell model with human adipose tissue–MSCs previously infected by the suicide gene HSV-Tk. The MSCs were good carriers of the gene for the treatment of U-87-derived glioblastoma. Kwon et al. [4] offered a combinatorial treatment based on MSCs against glioblastoma and to induce post nerve regeneration. Similarly to internalizing oncolytic viruses into MSCs, conjugating nanoparticles to MSCs can help in the accumulation of nanoparticles at tumor sites. When mediated by nanoparticles, MSCs can regenerate the damaged neurons in the central nervous system through the promotion of axon growth.

**Figure 1:**
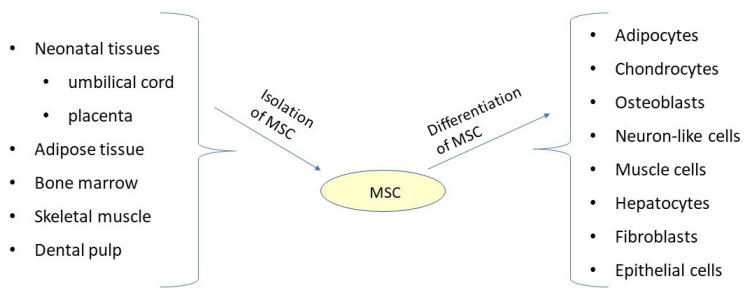
MSCs’ isolation from different neonatal and adult tissues, and in vitro differentiation into a wide variety of cell types.

It should be noted that therapy using MSCs is not restricted by histocompatibility, the formation of teratomas is not induced by MSCs, and ethical conflicts regarding the use of MSCs do not occur. MSCs have been isolated in bone marrow [5], adipose tissue [6,7,8,9], the umbilical cord [10,11], dental pulp [12,13], and the placenta [14,15] (Figure 1). In 2006, the International Society for Cellular Therapy established the minimum criteria to define MSCs based on the following biological characteristics: (a) adherent plastic cell growth under standard culture conditions, (b) positive expression of cell surface markers CD105, CD90, and CD73, (c) lack of expression of CD45, CD34, CD14 or CD11b, CD79alpha or CD19 and HLA-DR surface molecules, and (d) differentiation towards osteoblasts, adipocytes, and chondrocytes in vitro [16].

MSCs are relatively easy to isolate, culture, expand, and differentiate in vitro, making them excellent candidates for cell therapy [17,18,19,20,21,22]. MSC collection and isolation protocols are not standardized, making it difficult to compare studies to determine the precise mechanisms of MSCs’ migration. Continued research is needed to better understand the molecular mechanism of MSCs’ migration and to be able to use them for glioblastoma treatment [23].

MSCs modulate both innate and adaptive immune cells by disrupting their activation, proliferation, maturation, cytokine production, cytolytic activity, or antibody production. MSCs regulate inflammation by releasing anti-inflammatory cytokines (IL-4, IL-6, IL-10, IDO, PGE-2, and TGF-β) to trigger appropriate macrophage polarization. The immunosuppressive effects are related to the inhibition of T cell proliferation and the induction of regulatory T cells, thus promoting the transformation of macrophages from the anti-inflammatory M1 to M2 phenotype or contributing to immune homeostasis [24,25,26,27,28,29].

MSCs have tropism for glioma, which makes them potential vectors for the delivery of antitumor substances without affecting normal brain tissue. Glioma and MSCs have been shown to secrete various factors, such as stromal cell-derived factor 1 (SDF-1, also known as CXCL12), VEGF, PDFG, endothelial cell growth factor (EGF), TGF-β1, interleukin 8 (IL-8), and the MCP1 protein, which contribute to tumor tropism [30,31,32,33]. MSCs’ migration across the blood–brain barrier, which is similar to that carried out by leukocytes, has been proposed as a possible mechanism for MSCs’ tumor tropism. [30,34,35,36,37]. Furthermore, tumor tropism preferences vary depending on the MSC lineage. The endocrine signals of the tumor microenvironment influence the migration of MSCs, including mainly the regulatory signal produced by SDF-1/CXCR4 [35,38,39,40]. CXCR4 is a cell surface chemokine receptor that mediates cell dissemination, invasion, and proliferation present in tumor stem cells of a wide variety of tumors, such as glioma. There is a significant overexpression of CXCR4 in glioblastoma [41,42,43,44]. Cell migration is increased in the presence of growth factors, chemokines, p27, and matrix metalloproteases, while it is decreased in the presence of inhibitors of angiogenic signaling factors. The migration of MSCs obtained from the umbilical cord (UC-MSC) depends on angiogenic signaling factors and may share pathways with tumor angiogenesis. This could be an advantage for using MSCs against glioma [23,45,46,47].

## 2. Clinical and Therapeutic Use of MSCs

### 2.1. Therapeutic Gene Delivery

Gliomas can escape the immune system by secreting immunosuppressive agents, inhibiting T cell proliferation, and reducing immune responses. An immunotherapy strategy for treating gliomas is the administration of therapeutic genes to stimulate an immune response, e.g., genes that encode cytokines, such as interleukins (IL) and interferon (IFN) family genes [30]. Strategies for the delivery of genes or anticancer agents are based on the following: (a) augmentation gene therapy, which includes the expression of a gene to cause apoptosis, to improve the sensitivity of the tumor to chemotherapy or radiotherapy, or to introduce a tumor suppressor gene, (b) gene silencing therapy, based on the inhibition of the expression of an oncogene through the use of an antisense RNA or DNA, (c) suicide gene therapy, that consists of administering an enzyme that converts the non-toxic prodrug to a toxic one in the tumor site, and (d) immunogenic therapy, which increases the immunogenicity of tumor cells or tissues in such a way that it can stimulate the immune response against the tumor [48].

The most commonly used gene for therapeutic gene delivery is tumor necrosis factor-related apoptosis-inducing ligand (TRAIL). TRAIL belongs to the tumor necrosis factor (TNF) superfamily. Its therapeutic interest against tumors lies in the fact that it effectively induces apoptosis of tumor cells without producing toxicity to neighboring normal cells through the activation of the TNF/CD95L axis (extrinsic apoptosis pathway). Nevertheless, this type of therapy presents the problem that TRAIL has a short pharmacokinetic half-life after intravenous administration. The use of MSCs has been proposed as a TRAIL-directed and prolonged administration vector [49,50,51,52]. However, certain tumors, such as glioblastoma, are resistant to TRAIL-directed apoptosis. To solve this problem, a combined treatment of TRAIL with drugs that sensitize glioma cells to apoptosis induced by TRAIL or other agents (chemotherapeutics, radiotherapy, lipoxygenase, carbenoxolone) has been used [23].

### 2.2. Oncolytic Virus Delivery

Oncolytic viruses are genetically modified viruses that replicate within tumor cells. Viral infections caused by oncolytic viruses in tumor cells induce in situ cell lysis, which releases viral particles into neighboring tumor cells, resulting in further viral infections. The goal is to spread the virus throughout the tumor environment in such a way that after countless rounds of infections, the tumor is completely eradicated.

In 2017, a trial was carried out with the Zika virus [53] to test the effectiveness of using oncolytic viruses in therapy against glioblastoma. This is an RNA virus belonging to the flavivirus genus, and it is the causative agent of microcephaly. Zika virus presents great tropism for developing CNS cells, mainly for neural stem and progenitor cells. This property was tested to see whether it might be of benefit for a more effective treatment against glioblastoma. The study used patient-derived glioblastoma stem cells (GSCs), which express stem cell markers, have self-renewal capacity, have differentiation potential, and form tumors after xenotransplantation, and differentiated glioma cells (DGCs). Both cells were infected with two strains of the Zika virus, and after 48 h, an analysis through immunofluorescence microscopy showed that more than 60% of the GSCs were infected regardless of the type of strain. Furthermore, 90% of the infected GSCs presented the dedifferentiation marker SOX2. These data confirm the tropism of the Zika virus towards undifferentiated nerve cells. Furthermore, the study demonstrated that Zika virus (a) primarily infects human SOX2+ GSCs and inhibits proliferation in vitro, (b) causes loss of self-renewal and proliferation in glioblastoma organoids, (c) targets GSCs and, with less effects, DGCs and normal neuronal cells in human tissue samples, and (d) attenuates glioma growth, prolongs survival, and has marginal effects on normal neuronal cells [53].

The main limitation of treatment with oncolytic viruses is the cellular delivery system, as most of the systemically administered virus is eliminated through phagocytosis. This is where MSCs come into play as a secure management system. The internalization of oncolytic viruses within MSCs allows for their intravenous administration and safe delivery to tumor cells [4]. Human-bone-marrow-derived MSCs (BM-MSCs) have been used as oncolytic virus delivery vectors for the treatment of mice carrying the human U87 glioma cell line and ovarian tumors [23].

Another example in oncotherapy is the thymidine kinase gene of the herpes simplex virus (HSV-tk). Thymidine kinase is an anticancer, prodrug-converting enzyme. It converts ganciclovir into its toxic form, which inhibits DNA synthesis, and, consequently, cell death occurs. Administration of HSV-tk through MSCs obtained from BM-MSCs produces a more efficient distribution within tumors compared to injection of this gene in viral vectors. MSCs engineered with HSV-tk have recently been used in combination with ganciclovir in the treatment of C6 glioma and Panc02 pancreatic cancer [54]. Another study demonstrated the antitumor effect of MSCs obtained from adipose tissue (AT-MSC) transduced with the HSV-tk gene for the treatment of glioblastoma derived from the U87 cell line [3]. These results show that MSCs, regardless of their origin, are good carriers of suicide genes for glioblastoma gene therapy [55,56,57,58,59].

Oncolytic viruses have been used as effective weapons against glioblastoma. Clinical studies for the treatment of glioblastoma have included more than 20 oncolytic viruses that have been examined [60]. Herpes simplex virus-1 [61,62,63], adenovirus [64], reovirus [65], measles viruses [66], Newcastle disease viruses [67], and poliovirus [68] are a few of them. Nevertheless, there are various factors that complicate their effectiveness, such as low levels of viral transduction to glioblastoma cells, immunogenicity against viruses [69], or the dispersion of tumor cells forming metastatic niches. For this reason, delivery systems for oncolytic viruses to tumor cells have been devised [70], with MSCs as virus carrier cells that can reach places that viruses do not reach due to the dispersion of tumor cells and the immunological reaction against viruses [69].

Multiple types of viruses can be combined with MSCs. But, there are several points that should be considered when using this kind of therapy in order to improve the benefits of the combination [59,70,71,72]. First of all, viral infection of MSCs and viral replication into them is needed for an optimal tumor viral delivery; protection of virus recognition by the immune system should be guaranteed; MSCs should load their viruses near the tumor mass in such a way that a homogeneous production of new viral particles will be favored by the tumor mass; and MSCs should better keep their immunogenic capacity to promote an antitumor immune response.

### 2.3. miRNA Delivery

MicroRNAs (miRNAs) are small, non-coding RNAs that operate as negative post-transcriptional regulators of gene expression. They are an important tool to target mRNAs, and some of them are potent tumor suppressors [73,74,75,76,77,78,79]. It has been observed that miRNAs are abundant in extracellular exosomes secreted by a wide type of mammalian cells, including MSCs. Given the lability (they are easily degraded) of miRNAs, they cannot be injected directly, so the inclusion of therapeutic miRNAs in exosomes from MSCs may be a new treatment strategy against glioma [23]. Overexpression of miR-9 is associated with increased apoptosis in glioblastoma by negatively regulating the expression of the structural maintenance gene on chromosome 1 (SMC1A) in tumor cells. Therefore, those therapies that induce miR-9 expression in glioblastoma cells might have a positive therapeutic effect [79].

MSCs obtained from the umbilical cord (UC-MSC) secrete extracellular vesicles that contain miRNAs, whose complementary bases are found in the RNA of glioma cells, which inhibits cell proliferation and stimulates apoptosis. Various types of miRNAs have been found that act at different levels, including (a) blockade of the AKT-mTOR pathway and then inhibiting invasion and reducing the proliferation rate of glioblastoma, (b) inhibition of MET expression, which sensitizes the GSCs to ionizing radiation, (c) preventing the transformation of malignant astrocytes into GSCs, (d) negative regulation of Notch1, (e) suppression of cyclin D1 expression, (f) downregulation of NF-kB through the negative regulation of the inhibitor of the epsilon subunit of nuclear factor kappa-B kinase (IKBKE), (g) negative regulation of the expression of cyclin B1, (h) negative regulation of B-cell lymphoma protein 2 (Bcl-2), an antiapoptotic protein, allowing glioblastoma to be sensitized to radiation, (i) blockade of the MAPK pathway, which is responsible for regulating cell apoptosis, proliferation, and resistance to chemotherapy, (j) blockade of MDM2, which is responsible for the degradation of the tumor suppressor protein p53, (k) blockade of the activity of TGF-β, a cytokine associated with proliferation, angiogenesis, invasion, and immunosuppression, and (l) inactivation of MGMT, a protein responsible for repairing DNA damage, which sensitizes glioblastomas to temozolomide [80].

The use of MSC-derived exosomes for the delivery of therapeutic miRNAs for cancer therapy seems to be controversial nowadays, as, in some cases, those miRNAs have contributed to increased cancer phenotypes, as in osteosarcoma, via miR-208a [81], in multiple myeloma, via miR-146a [82], in gastric cancer, via miRNA-221 [83], in glioma cells, via miR-1587 [84], and even in breast cancer cells, through miR-23b expression, which acquired a dormant phenotype in metastatic niches [85].

Also, fortunately, research has demonstrated that various tumors seem to be reduced when the delivery of miRNAs from MSC-derived exosomes is applied, as in hepatocellular carcinoma, as miR-122 improves drug sensitivity [86] and microRNA-15a reduces hepatocellular carcinoma progression via downregulation of SALL4 [83], in non-small-cell lung cancer cells through microRNA-193a, which reduces cisplatin resistance via targeting LRRC1 [87], in pancreatic cancer, via miRNA-1231 [88], and in glioma, as microRNA-133b suppresses glioma progression via the Wnt/β-catenin signaling pathway by targeting EZH2 [89].

## 3. Methods to Improve MSCs’ Tropism

MSCs’ tumor tropism due to the chemokines secreted by tumor cells is a key characteristic that permits the administration of specific therapeutic agents. However, drugs incorporated into the cytoplasm of MSCs have reduced viability and migratory capacity, in addition to having a limited loading capacity. Therefore, to improve the effectiveness of MSC-based therapy against cancer, methods must be sought to overcome these limitations [90]. The use of CAR-T cells [91,92,93] or conjugation with nanoparticles [94,95,96,97,98] are two potential ways to improve the tropism of MSCs.

### 3.1. CAR-MSC Cells

Adoptive immunotherapy of T cells genetically modified to express chimeric antigen receptors (CARs) has been established as a promising approach for the treatment of glioblastoma. The therapeutic interest in CAR-T cells focuses on their great ability to specifically target a tumor antigen and avoid the need for antigen presentation by the major histocompatibility complex. Several CAR antigens are currently in clinical trials for glioblastoma, including epidermal growth factor receptor variant III (EGFRvIII), human epidermal growth factor receptor 2 (HER2), and interleukin 13Ra2 receptor (IL-13Ra2) [99].

**Figure 2:**
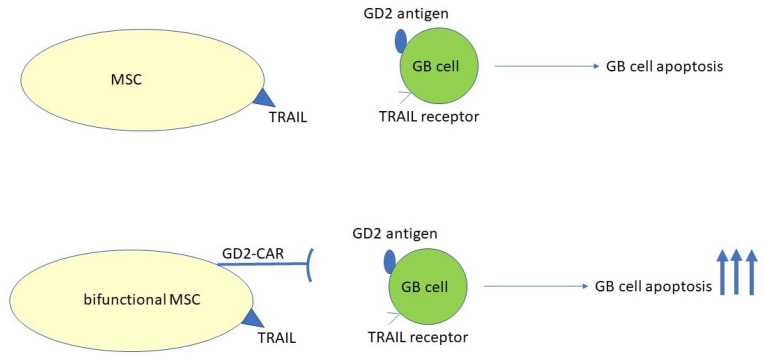
Use of chimeric antigen receptors (CARs) to increase tropism of MSCs. Engineered bifunctional MSCs have more tropism toward glioblastoma cells. The interaction of TRAIL and TRAIL receptor induces apoptosis of tumor cells. However, this action is more selective when a GD2-CAR is produced by MSCs and binds the GD2 antigen expressed by tumor cells, thus enhancing apoptosis.

Neuroectoderm-derived neoplasms, such as glioblastoma, sarcomas, and neuroblastoma, express high levels of the disialoganglioside GD2 antigen. Taking this into account, a bifunctional MSC was designed that simultaneously expressed anti-GD2-CAR, to improve tropism for the tumor cell, and TRAIL, which is a therapeutic molecule that will destroy tumor cells. The results obtained demonstrated that bifunctional MSCs had greater destruction capacity than MSCs with TRAIL alone [100] (Figure 2).

### 3.2. Conjugation of MSCs with Nanoparticles

Nanometals are characterized by having a small size, a large surface area compared to their volume, the possibility of fixing different molecules on their surface, the ability to cross cellular and/or tissue barriers, and a long circulation time in the bloodstream. These characteristics make them suitable for clinical use as drug delivery systems or agents for targeting tumor cells [101,102].

**Figure 3:**
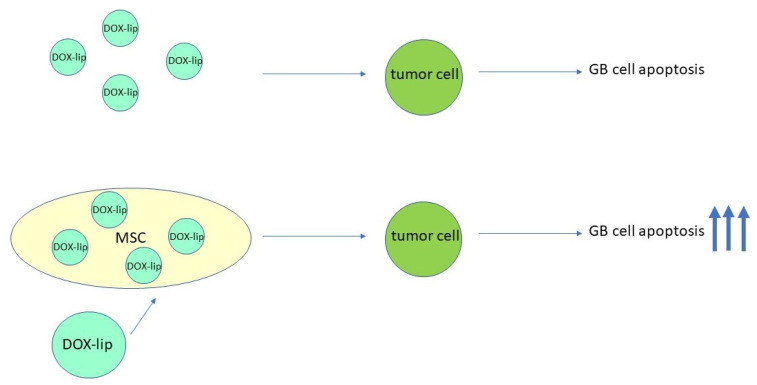
Use of DOX-Lips-loaded MSCs to increase the efficacy of antitumor treatment. Dox-Lips are liposomes that contain the antitumor agent doxorubicin. DOX-Lips alone can kill some tumor cells, but the efficacy of DOX-Lips is enhanced when they are delivered to tumor cells via MSCs.

Internalization of drugs in MSCs is limited, and this reduces their viability and migratory capacity. A strategy to expand the carrying capacity of MSCs is to modify their cell surface. A study conducted by Takayama et al. [90] determined the influence of these modifications on the characteristic properties of MSCs, such as migration. In that study, the MSC cell line C3H10T1/2 was functionalized with the anticancer agent doxorubicin (DOX) encapsulated within liposomes (DOX-Lips) for higher drug loading through the avidin–biotin complex method (Figure 3). Next, different parameters were evaluated, including the amount of DOX in DOX-Lip-C3H10T1/2 cells, the influence of DOX on C3H10T1/2 cells, and the antitumor effect of DOX-Lip-C3H10T1/2 in vitro on the murine colon adenocarcinoma cell line and in vivo on mouse models carrying subcutaneous tumors and lung metastases. The results were promising, as cell surface modification with DOX-Lips using the avidin–biotin complex method did not affect the proliferation, attachment, migration, or tumor localization ability of C3H10T1/2 cells. Furthermore, it produced a great antitumor effect on Colon26/GFP cells. Tumor cells treated with DOX-Lips-C3H10T1/2 cells had a lower percentage of viability compared to unmodified C2H10T1/2 at 48% and 89%, respectively. It was also possible to determine that endocytosis is the mechanism through which tumor cells incorporate the drug [90].

## 4. Exosomes Derived from MSCs

The secretome of MSCs is formed by a set of proteins expressed by MSCs and secreted into the extracellular space. Cytokines, chemokines, growth factors, and extracellular vesicles (EV) belong to this group. The latter, depending on its size and origin, is subdivided into ectosomes and exosomes. Ectosomes are vesicles of 50 nm to 1 μm in diameter generated by direct budding from the plasma membrane, while exosomes are vesicles of 40 to 160 nm that originate from endosomal compartments, and they are ubiquitous in body fluids. Exosomes are made up of a lipid bilayer membrane and can house molecular components, such as DNA, RNA, and proteins. The interest that exosomes have aroused in the clinic is due to the fact that they are capable of influencing various activities through the exchange of bioactive components both with neighboring cells and with distal cells. MSCs present different characteristics than exosomes derived from MSCs [103,104,105,106,107,108,109,110,111].

The biogenesis of exosomes involves the formation of intracellular multivesicular bodies (MVBs) that can follow two paths. They can fuse with lysosome to be degraded, or they can fuse with the plasma membrane to release their cargo into the extracellular space (exosomes). The content of MSC-derived exosomes consists of (a) proteins that control cell growth, proliferation, adhesion, migration, and morphogenesis capabilities of MSCs; (b) RNA involved in the regulation of cell survival, cell differentiation, and the modulation of the immune system; and (c) DNA [103].

Cell–cell contact between MSCs and glioblastoma cells generates a unique secretome, which could be related to the significant increase in the tumorigenic properties of glioblastoma cells [112,113,114]. However, like MSCs, exosomes derived from MSCs can present pro-tumorigenic or anti-tumorigenic effects in the various processes in which they intervene (tumor growth, angiogenesis, metastasis, and drug resistance) depending on their origin [103]. In glioblastoma, internalization of exosomes derived from AT-MSCs stimulated cell proliferation, while internalization of exosomes from BM-MSCs and UC-MSCs inhibited proliferation and induced apoptosis [112].

## 5. MSCs Associated with Gliomas

Glioma-associated MSCs (gbMSCs), isolated for the first time in 2014 [115], are capable of secreting different factors depending on the conditions in which they are found, e.g., hypoxia. They are characterized by the positive expression of CD105, CD90, and CD73 and negative expression of CD14, CD31, and CD45 [116]. A study by Svensson et al. [117] identified two subpopulations, CD90+ gbMSC and CD90- gbMSC. CD90- gbMSCs produce higher levels of PGE2 and VEGF than CD90+ gbMSCs. It is known that PGE2 induces immunosuppression and that VEGF is a factor that favors greater tumor angiogenesis in addition to greater recruitment and proliferation of MSCs in glioma. This indicates that the CD90- gbMSC subpopulation plays a very important role in tumor vascularization and immunosuppression, and it is also capable of differentiating into pericytes and further contributing to neovascularization in the glioma microenvironment. Therefore, a higher percentage of gbMSCs, whether CD90+ or CD90-, is associated with a worse survival rate [117]. Various studies suggest the promoting role of gbMSCs in the aggression and progression of gliomas [30].

Shahar et al. [118] demonstrated that glioma-associated human MSCs (GA-hMSCs) are present in high-grade human gliomas although they are not tumorigenic; instead, they are capable of enhancing the proliferation of glioma-initiating cells, which are responsible for tumor recurrence. Also, a greater presence of GA-hMSCs is associated with a worse prognosis and survival [118]. These findings raise an important question regarding whether the use of MSCs as a treatment for glioblastoma could worsen the patient’s condition.

## 6. Do MSCs Support or Suppress Tumor Progression of Gliomas?

Once MSCs are localized in the tumor microenvironment, they can interact with tumor cells and, as a result, secrete a wide range of cytokines and growth factors that can contribute to cell survival, growth, motility, and immune escape of tumor cells [112]. However, various studies have demonstrated different results of the potential use of MSCs in therapy. Some indicate that MSCs can facilitate tumor progression by reducing apoptosis and promoting epithelial-to-mesenchymal transition, while others can inhibit it by exerting an immunosuppressive effect.

### 6.1. BM-MSC

Bone-marrow-derived MSCs (BM-MSCs) can negatively affect tumor angiogenesis through the release of antiangiogenic factors [23]. Furthermore, it has been shown that BM-MSCs promote senescence of the U87 glioblastoma cell line by inducing changes in cell morphology and by increasing the production of cytokines (IL-6, IL-8, leukemia inhibitory factor (LIF), CCL2/MCP-1, and CXCL2) [119]. But, another study showed that BM-MSCs can enhance the invasive capacity of the glioblastoma cell line U373 and inhibit it in U87. This seems to be due to the fact that there are significant differences in the gene expression profiles of the cell lines, as the first cell line presents more mesenchymal characteristics (associated with malignancy) than the second one [120]. It has also been seen that BM-MSC-conditioned medium inhibits the proliferation of the C6 glioma cell line but promotes its migration and invasion [121].

### 6.2. UC-MSC

Umbilical-cord-derived MSCs (UC-MSCs) have been shown to inhibit the growth of the U87 glioblastoma cell line by suppressing angiogenesis and promoting apoptosis in the tumor microenvironment [23]. The antitumor function of human UC-MSCs occurs through the positive regulation of PTEN in glioma cells (SNB19, U251, 4910, and 5310) [23,122]. Akt regulates the function of proteins involved in the cell cycle, proliferation, apoptosis, and invasion, all of which are important in tumorigenesis. Akt is overactivated in many glioblastomas due to loss of PTEN function. Overexpression of PTEN negatively disrupts the PI3K/Akt signaling pathway, resulting in decreased tumor cell growth and migration. Furthermore, UC-MSCs can induce apoptosis through downregulation of the X-linked inhibitor of apoptosis (XIAP). Overexpression of XIAP is a mechanism through which tumor cells acquire resistance to apoptosis; if its expression decreases, apoptosis is favored. Likewise, UC-MSCs induce the activation of TRAIL, which ultimately induces apoptosis [122].

### 6.3. AT-MSC

Adipose-tissue-derived MSCs (AT-MSCs) can increase the size of glioma tissue by reducing apoptosis and VEGF secretion, in addition to promoting epithelial-to-mesenchymal transition in glioma cells. On the other hand, AT-MSCs can have an inhibitory effect against the 8MGBA glioblastoma cell line due to the synergistic action of the soluble factors it releases, which include IL-6, IFN-γ, and G-CSF [123].

AT-MSCs are involved in promoting the malignant phenotype of several tumors, like cervical cancer [124], breast cancer [125], colon cancer [126], and ovarian tumors [127]. Even promotion of tumorspheres [125] and angiogenesis [128] has been documented in breast cancer.

On the contrary, Kucerova et al. [129] have documented AT-MSC-mediated prodrug cancer gene therapy. Second, the ability to inhibit cell growth and to induce apoptosis of primary ovarian carcinoma after exposure of cells to microvesicles derived from human immortalized AT-MSCs has been determined [130]. Third, AT-MSCs cultured at high density express IFN-β and TRAIL and suppress the growth of H460 human lung cancer cells [131]. Fourth, AT-MSCs enhanced the effects of radiotherapy on hepatocellular carcinoma [132]. Fifth, the therapeutic potential of AT-MSCs as cellular vehicles for prodrug gene therapy against brainstem gliomas is now clear [133]. Lastly, AT- and BM-derived MSCs appear to have similar tumor tropism in vitro. Given the feasibility of obtaining larger numbers of AT-MSCs from adipose tissue under local anesthesia, adipose tissue might be a more efficient source of MSCs for research and clinical applications [6].

## 7. Conclusions

MSCs are multipotent cells capable of differentiating into various cell types. They have the ability to migrate to the tumor area and integrate into tumor vessels [3]. All of this, added to their easy obtaining, makes them suitable for cell therapy. However, the lack of a standardized protocol has made it difficult to compare results. Therefore, one of the aspects to consider is the establishment of a universal protocol that allows for obtaining more homogeneous and representative data.

The use of MSCs in cancer cell therapy presents divergence in results. On the one hand, it shows antitumor properties that could improve the survival rate and quality of the patient’s life. On the other hand, it shows pro-tumor properties that could facilitate tumor progression. So, a question to ask is to what extent the use of MSCs in the treatment of glioblastoma is viable. Because most research has been carried out in in vitro cells and small experimental animals, more experiments are required to move to the next level, including experimentation with large animals and, finally, a clinical trial in humans, as long as the benefits outweigh the risks. Regarding the differences found in the different types of MSCs, UC-MSCs seem to present better antitumor properties than AT-MSCs and BM-MSCs. However, it must always be considered that the properties of MSCs vary depending on the cell type from which they are obtained, as well as the tumor selected for MSC treatment. Another strategy that is recently gaining relevance is the use of exosomes derived from MSCs. These have the same characteristics as MSCs but have the advantage that they are not immunogenic and can be produced on a large scale for clinical application at a low cost [103].

MSCs promote or suppress the growth of glioma cells depending on their origin and the conditions in which they are found. For example, UC-MSCs have greater proliferation and expansion potential than BM-MSCs [119]. These results highlight the biological complexity that MSCs present for their use as administration vectors and the need for more in-depth research to better understand their possible effects, whether beneficial or harmful. Therefore, it is essential to consider various factors, such as the type of tumor to which MSCs will be administered, heterogeneity, the tumor microenvironment, and the sources of MSCs before using them in clinical therapy [23].

In short, current glioblastoma therapy is not a curative treatment but rather palliative; that is, it seeks to reduce symptoms and thus improve the person’s quality of life. For this reason, various alternative treatment routes have emerged, including antiangiogenic drugs, the use of monoclonal antibodies, nanoparticles loaded with drugs, or the use of MSCs, the latter being the ones that have shown the best results. In the future, it would be interesting to determine whether the combination of different treatments would lead to an improvement in the antitumor response. This requires more research to elucidate the complex molecular mechanisms by which glioblastomas are capable of developing great resistance to treatments, as well as the effect that each line of treatment would have on the tumor microenvironment individually and in combination.
